# A New Approach for On-Chip Production of Biological Microgels Using Photochemical Cross-Linking

**DOI:** 10.1021/acs.analchem.4c01574

**Published:** 2024-06-11

**Authors:** Francesco Del Giudice, Dan J. Curtis, Anders Aufderhorst-Roberts

**Affiliations:** † Complex Fluids Research Group, Department of Chemical Engineering, School of Engineering and Applied Science, Faculty of Science and Engineering, Swansea University, Swansea SA1 8EN, United Kingdom; ‡ Centre for Materials Physics, Department of Physics, Durham University, Durham DH1 3LE, United Kingdom

## Abstract

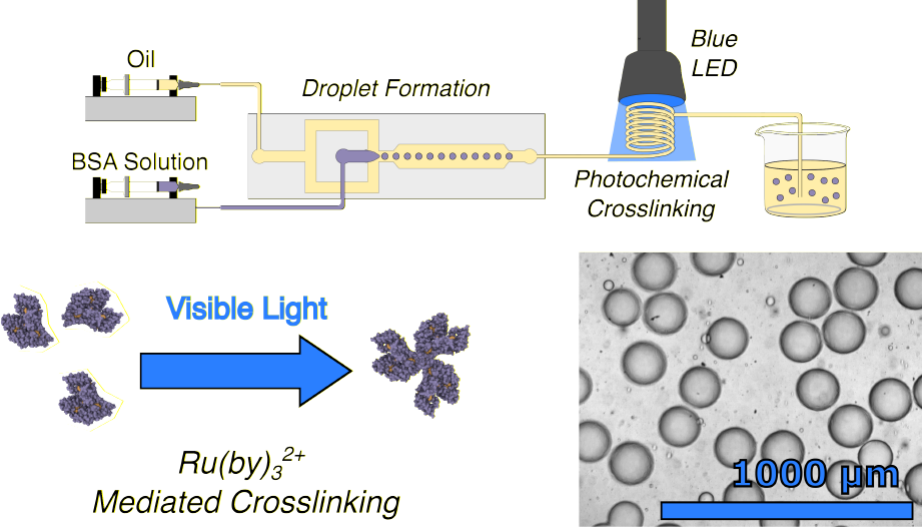

Photochemical cross-linking is a key step for manufacturing microgels in numerous applications, including drug delivery, tissue engineering, material production, and wound healing. Existing photochemical cross-linking techniques in microfluidic devices rely on UV curing, which can cause cell and DNA damage. We address this challenge by developing a microfluidic workflow for producing microgels using visible light-driven photochemical cross-linking of aqueous droplets dispersed in a continuous oil phase. We report a proof-of-concept to construct microgels from the protein Bovine Serum Albumin (BSA) with [Ru(bpy)_3_]^2+^ mediated cross-linking. By controlling the capillary number of the continuous and dispersed phases, the volumetric flow rate, and the photochemical reaction time within the microfluidic tubing, we demonstrate the construction of protein microgels with controllable and uniform dimensions. Our technique can, in principle, be applied to a wide range of different proteins with biological and responsive properties. This work therefore bridges the gap between hydrogel manufacturing using visible light and microfluidic microgel templating, facilitating numerous biomedical applications.

## Introduction

Microgels are structured, microscopic, cross-linked polymeric networks swollen by the solvent in which they are dissolved.^1^ They are desirable in numerous applications, including drug delivery,^2^ wound healing,^3^ tissue engineering,^4^ encapsulation,^5^ and the food industry.^6^ A key scientific challenge in using microgels in the biomedical context is the need for the microgel material to be nontoxic and biocompatible.^7^

Microfluidic technologies are an increasingly desirable approach for microgel manufacturing, because they can generate microgels with a uniform size distribution.^8^ Such approaches typically involve a microfluidic setup, where nonmiscible liquids meet at a junction to generate uniform droplets.^9^ Cross-linking occurs inside the droplet, either by the diffusion of cross-linking chemicals or through photochemical reactions triggered by external illumination. The latter has the advantage that cross-linking is independent of droplet formation, allowing independent control of microgel size and shape, since microgel cross-linking does not rely on the volumetric flow rates of the reagents. However, existing photochemical cross-linking techniques in microfluidic devices rely on UV curing,^9^ which can cause cell and DNA damage.^10^ Manufacturing biocompatible microgels without UV light remains problematic because of the trade-off between the biocompatibility of the cross-linking agent and the wavelength required to enable the cross-linking, which is generally in the UV spectrum.

In this work, we address this challenge by developing a microfluidic workflow for producing microgels using photochemical cross-linking with visible light. We provide a proof-of-concept based on the production of microgels from Bovine Serum Albumin (BSA), a globular protein popular for a range of biochemical assays due to its stability, low cost, and low reactivity.^11^ We cross-link our BSA microgels with a ruthenium cross-linking strategy.^12^ This strategy uses visible light photochemical cross-linking, with a high absorptivity,^13^ thus allowing cross-linking at low concentrations of cross-linker and avoiding the unwanted consequences of UV light irradiation.

## Materials and Methods

### Material Preparation and Characterization

BSA (Sigma-Aldrich, UK) was suspended in deionized water and placed on a roller mixer for a minimum of 1 h to enable complete dissolution of protein. Protein concentration was measured by absorption at 280 nm, using an extinction coefficient of 43 824 M^–1^ cm^–1^. The final suspension was then diluted and mixed with concentrated sodium phosphate buffer prepared at pH 7.4, tris(2,2′-bipyridyl)-dichlororuthenium(II) hexahydrate (Ru(BiPy)_3_), and sodium persulfate (NaPS) directly before use, with final concentrations of 100 mg/mL BSA, 25 mM sodium phosphate, 100 μM Ru(BiPy)_3_, and 10 mM NaPS.

Mineral oil (Sigma-Aldrich, UK) with the addition of 1 wt % Span 80 (Sigma-Aldrich, UK) was employed as a continuous phase liquid, in agreement with previous work.^14^ The interfacial tension between the dispersed and continuous phases was measured using a force tensiometer (Sigma702, Biolin Scientific) equipped with a du Nouy ring. We evaluated the density of each liquid using a calibrated pipette (Gilson, UK) and a scale with 0.1 mg accuracy, obtaining the values of density for the BSA, the BSA with cross-linker, and the mineral oil with Span 80 equal to ρ_BSA_ = 0.991 g/mL, ρ_BSA-cross_ = 0.997 g/mL, and ρ_oil_ = 0.779 g/mL, respectively. We measured interfacial tension values γ_BSA–oil_ = 4.33 ± 0.1 mN/m and γ_BSA-cross–oil_ = 3.42 ± 0.01 mN/m, for BSA and oil, and BSA-cross-link and oil, respectively.

The viscosity curves for BSA (Figure S1) were measured using an Anton Paar MCR702 rheometer with a cone and plate configuration (60 mm, 1° angle). The viscosity of the mineral oil was equal to 29 mPa s.^15^ Time scales for bulk gelation were measured using a Netzsch Kinexus Pro rheometer equipped with a 20 mm steel plate and plate configuration in which the lower plate was replaced with a custom-built photodiode module, as previously reported.^16^

#### Microfluidic Apparatus

The BSA droplets were generated within commercial flow-focusing glass microfluidic devices (Dolomite Microfluidics, UK) having three etching depths, namely, 100 μm (part 3000301), 190 μm (part 3000347), and 275 μm (part 3200823), with hydrophobic coating on their surfaces. The flow of the two phases was controlled using two independent pumps (KD Scientific). The samples were loaded in glass syringes (Hamilton Glass) with volumes of 10 mL (continuous phase) and 1 mL (dispersed phase). The connections between the microfluidic device and the syringe pumps were commercially available tubing with an internal diameter of 800 μm and an external diameter of 1.6 mm (Dolomite Microfluidics). Droplet formation was observed using an inverted microscope (Zeiss, Axiovert 135) connected to a high-speed camera (Photron Mini-Ux 50) acquiring videos at 500–2000 fps. Droplet length, height, and generation frequency were manually calculated from the experimental videos.

#### On-Chip Gelation

Sample gelation was initiated photochemically through illumination with a 460 nm light emitting diode as previously described.^16^ Based on the same work,^16^ the illumination intensity was set to 40 mW cm^–2^ at 452 nm.

## Results and Discussion

### Design Principle and Experimental Approach

**Figure 1:**
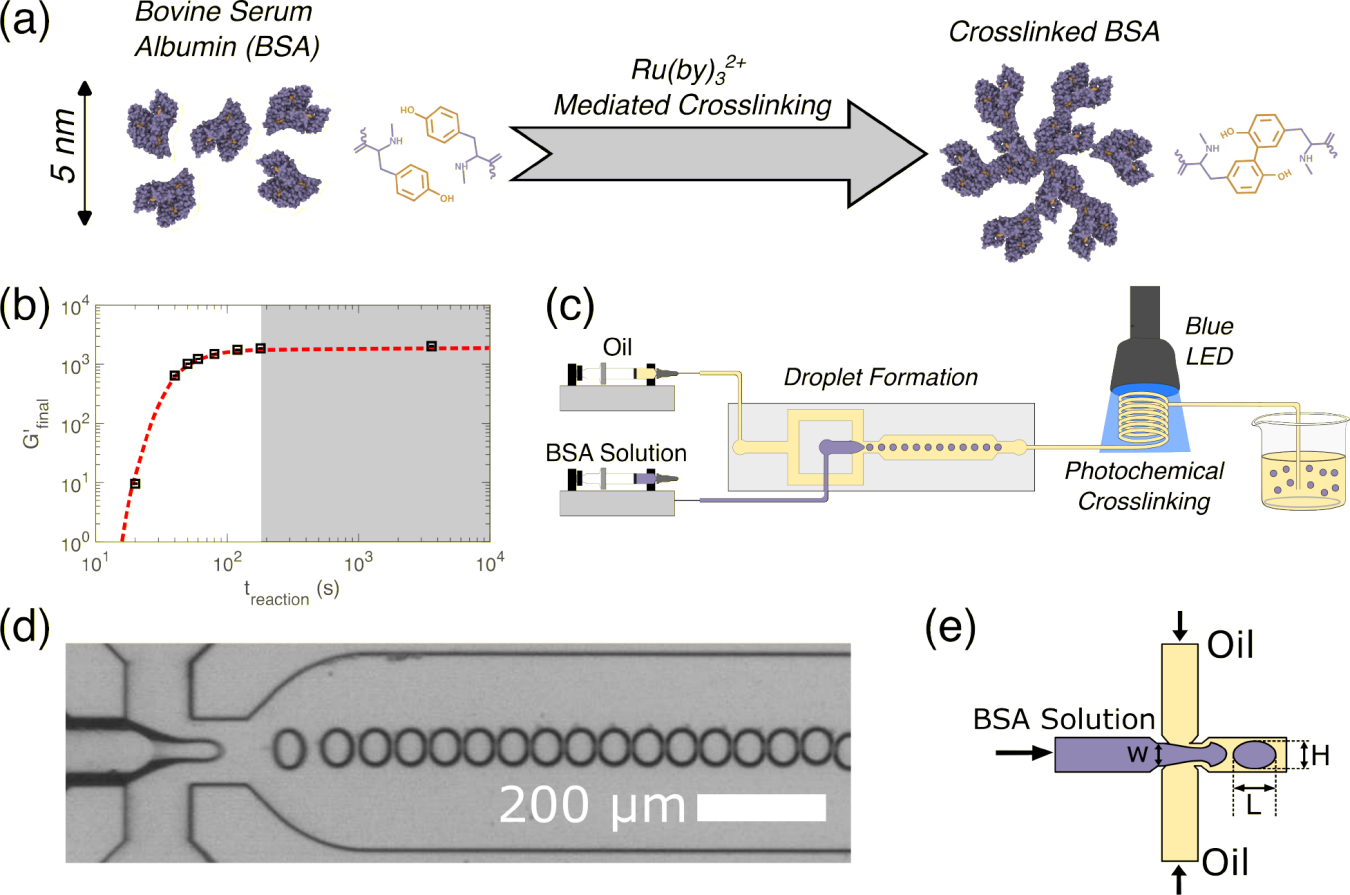
(a) Schematic showing the photochemical cross-linking reaction of Bovine Serum Albumin (BSA) through the formation of covalent dityrosine bonds. (b) The bulk elastic modulus of a BSA hydrogel evolves with photochemical cross-linking time, showing that reaction times exceeding 3 min (shaded region) result in no further change in final modulus (dashed line is a guide for the eye). (c) Droplets of BSA solution are generated in a continuous oil phase through a commercial microfluidic flow-focusing device, photochemically cross-linked within a coiled capillary and collected in a beaker containing the continuous oil phase. (d) Experimental snapshot of the generation of droplets in a flow-focusing microfluidic device. (e) Detailed schematic of flow-focusing shows the formation of droplet with length *L* and height *H*.

We cross-linked BSA into hydrogel particles using the [Ru(bpy)_3_]^2+^ strategy.^12^ The mechanism that underlines this strategy involves using visible light to generate a ruthenium-based radical. This leads to the radicalization of cysteine and tyrosine residues on the solvent-exposed surface of the protein,^17^ in the presence of sodium persulfate, an electron acceptor (Figure 1a). This results in the formation of a permanent dityrosine cross-link between adjacent proteins.

The [Ru(bpy)_3_]^2+^ cross-linking strategy is itself attractive as it has been shown to reach higher penetration depths beneath the surface of the material and is nontoxic, making it attractive for achieving complete and rapid cross-linking.^18^ It is one of the most commonly used strategies for protein hydrogel formation^16,19−21^ and is increasingly being investigated for biomedical applications, including cell culture^18^ and tissue sealants.^22^

Rheological measurements of the time scales of gelation of bulk BSA samples (Figure S2) showed an increase in the storage modulus as a function of the reaction time, following an initial delay time of approximately 30 s, which is consistent with previous work.^16^ The cross-linking was fully completed within 3 min, as the variation in *G*′ did not exceed 10% after this point (Figure 1b). This time scale corresponds to a structural length scale of 400 μm, which is the spacing between the rheometer plates and is of the same order of magnitude as the microgels prepared in this study.

We designed a microfluidic workflow to generate BSA microgels of different sizes (Figure 1c). The two syringe pumps were loaded with a dispersed phase (the material forming the droplet) of BSA solution with cross-linking reagents and a continuous phase (the fluid surrounding the droplet) of mineral oil with 1 wt % Span 80 surfactant. Monodisperse droplets (Figure 1d) were generated by passing both fluids through a flow-focusing droplet generation device (Figure 1e).

Following the droplet generation step, we then employed a 550 mm long tube with an internal diameter of 800 μm that we folded into a coil exposed to a blue LED light mounted on a fixed stand (Figure 1c) with a peak emission wavelength of 460 nm, which is close to the 452 nm maximum absorbance wavelength of the (Ru(BiPy)_3_) cross-linking agent.^12^

We used a volumetric flow rate of the dispersed phase *Q*_d_ = 5 μL/min, and of continuous phase *Q*_c_ = 40 μL/min, thus providing an overall volumetric flow rate *Q*_tot_ = 45 μL/min. The two values of the volumetric flow rate were chosen arbitrarily based on the results without the cross-linker. By keeping the overall flow rate constant, we could obtain microgels having different sizes depending on the etching depth of the microfluidic device employed. The coiled tubing exposed to the visible light included 350 mm of the tubing with an overall volume *V* ≈ 176 μL. The residence time of the microgels in the coiled tube was calculated as τ = *V*/*Q*_tot_ ≈ 4 min. The ratio between the residence time and the gelation time is known as the Damkohler number,^23^*Da*. Assuming a reaction completion time of *t*_reaction_ = 3 min (Figure 1b), our workflow operates at *Da* = τ/*t*_reaction_ = 1.33, which signifies that there was sufficient time to complete the cross-linking in the tube. A value of *Da* ≥ 1 enables additional tolerance in line with standard engineering practice^24^ and would also be desirable for biomedical applications, because the sodium persulfate which mediates the photochemical cross-linking is toxic and remains in the sample if the reaction is incomplete.^25^ Clearly, changing the value of *Q*_tot_ would require adjustment of the tube length to maintain the requirement of *Da* ≥ 1.

After the cross-linking, we collected the microgels in an 8 mL glass vial containing 2 mL of mineral oil with 1 wt % of Span 80, constantly stirred by a magnetic stirrer to prevent aggregation of the microgels entering the vial from the tubing. The outlet of the microfluidic tubing was aligned with the direction of stirring to minimize the accumulation of microgel particles directly at the outlet and thereby prevent clogging. The rotation speed was kept low to avoid breaking the microgels. The collection step lasted around 20 min, after which the microgels were collected for further video analysis.

To guarantee the reproducibility of the experiments, we developed a well-defined experimental protocol described as follows. We first established a steady flow rate of the two streams and then waited 5 min to stabilize the flow within the microfluidic device while keeping the visible LED lamp turned off, thus preventing the cross-linking of any residue previously present in the photochemical cross-linking area of the setup. Additionally, we very briefly turned on the microscope light to ensure the droplet generation was stable over time. After this step, we turned on the visible LED lamp and waited 6 min for the generated droplets to cross-link fully, which corresponds to the time taken for the droplets to pass through the whole 550 mm long tube, including the section exposed to the visible light and the remaining tube connections.

### Microfluidic Experiments

**Figure 2:**
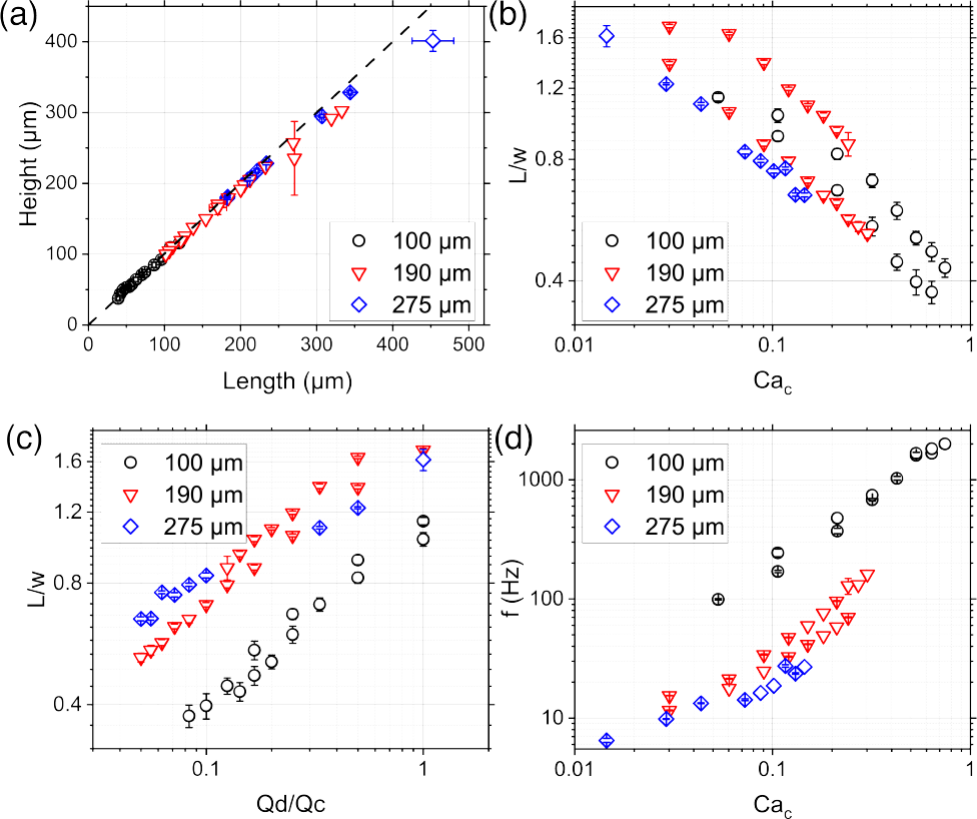
(a) Droplet height as a function of the droplet length for the three microfluidic devices employed in this work. The dashed line has a slope equal to 1 and an intercept equal to zero. The distribution of the data indicates that the generated droplets are mainly spherical. (b,c) Droplet length *L* normalized by the width of the droplet generation area *w* as a function of the Capillary number of the continuous phase *Ca*_c_ (b) and the ratio between the volumetric flow rate of the dispersed and the continuous phases (c). (d) Generation frequency *f* as a function of *Ca*_c_.

The generation of droplets in a flow-focusing device depends upon several experimental parameters, including the device geometry, the volumetric flow rate, and the physical properties of the two phases.^26^ For this reason, we first performed an experimental campaign to evaluate the required experimental conditions to achieve spherical droplets of a given size and generation rate (Figure 2). We worked with *Q*_c_ ≥ *Q*_d_, as this facilitated the production of spherical droplets (Figure 2a).

We observed that the droplet length *L* normalized by the width of the droplet generation area *w* was inversely proportional to the capillary number of the continued phase, defined as *Ca*_c_ = μ_c_*U*_c_/γ, where μ_c_ is the shear viscosity, γ is the interfacial tension, and *U*_c_ is the velocity of the continuous phase calculated as *U*_c_ = *Q*_c_/*hw*, where *h* is the height of the microfluidic device (Figure 2b). When increasing the value of *Ca*_c_, which practically meant an increase of *Q*_c_ in each microfluidic device, the droplet size decreased, in agreement with previous observations.^27,28^ Similarly, we observed that an increase in the flow rate ratio *Q*_d_/*Q*_c_ resulted in the formation of elongated droplets (Figure 2c). This behavior is because larger *Q*_d_ values require larger values of *Q*_c_ to keep the droplet size constant.^27^ We also observed an increase in the droplet generation frequency when increasing *Ca*_c_ (Figure 2d), in agreement with previous studies.^14,29^ By combining the results in Figure 2b and d, we can conclude that an increase in *Ca*_c_ resulted in the production of small droplets with a large generation rate, and vice versa.

**Figure 3:**
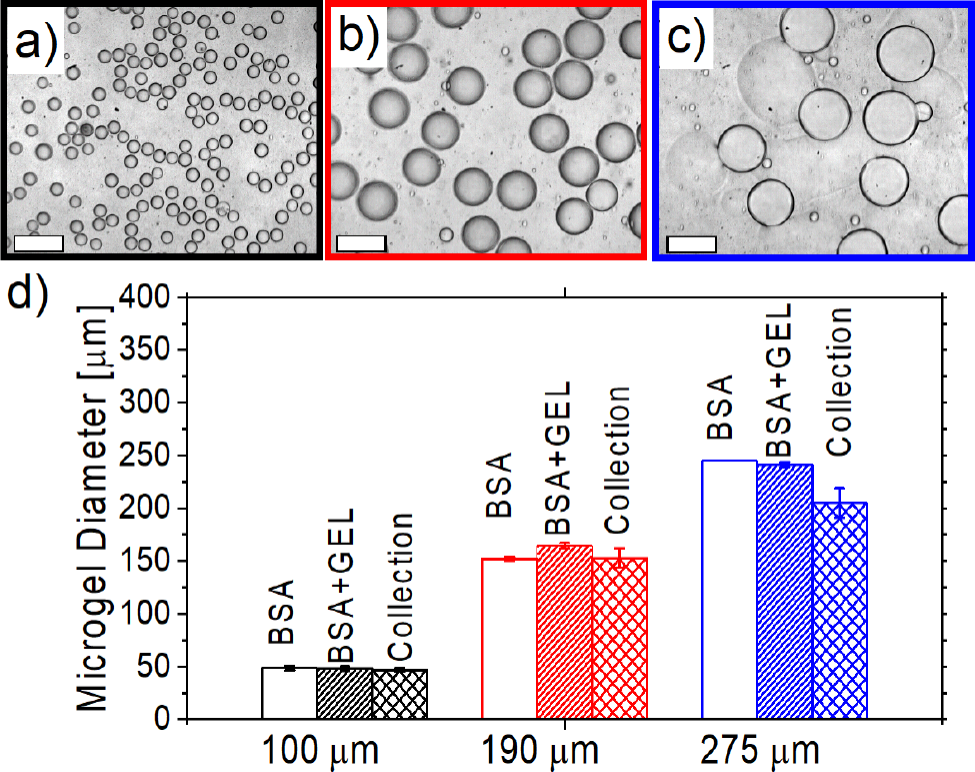
Experimental snapshots of microgels collected after photochemical cross-linking and manufactured using the 100 μm etched (a), the 190 μm etched (b), and the 276 μm etched microfluidic devices (c). The scale bar is 200 μm. (d) Comparison between the diameters of BSA droplets, BSA-cross-link droplets, and the collected microgel.

We manufactured spherical microgels by simply replacing the BSA with a dispersed phase containing the BSA and the cross-linker (Figure 3a–c), obtaining spherical and relatively monodisperse microgels by fixing *Q*_d_ = 5 μL/min and *Q*_c_ = 40 μL/min.

For each of the microfluidic devices, we then compared the size of the BSA droplets calculated directly after the droplet generation area with (i) the droplets obtained using BSA and cross-linker as the dispersed phase, and (ii) the size of the microgel after photochemical cross-linking and collection (Figure 3d). Good agreement was observed, which was expected because the main difference in observed droplet size could have resulted from the different values of interfacial tension, with γ_BSA–oil_ being roughly 25% larger than γ_BSA-cross-link–oil_. However, the droplet size is relatively weakly dependent on *Ca*_c_ in the range of Capillary number investigated (Figure 2b), meaning that the difference in γ values was expected to display a negligible impact on droplet size, as was observed in our experiments (Figure 3d). We also compared the size of the droplets before photochemical cross-linking and after collection, finding only minor deviations (Figure 3d). Taken together, our results showed that we could produce spherical BSA droplets across the three different microfluidic devices. We observed that an increase in the Capillary number of the continuous phase *Ca*_c_ led to the formation of smaller droplets and a larger droplet generation rate. We also observed that the microgels retained their shape and size during photochemical cross-linking.

Our workflow enabled the production of BSA-based microgels without the need to employ UV light. We were inspired by the work of Rapp et al.,^30^ which used a 4-inlet microfluidic system, which is more complex than our workflow. Another attempt in this direction has been made by Hu et al.,^31^ who employed capillary microfluidics to generate droplets using visible light. However, their approach relied on the Belousov–Zhabotinsky (BZ) reaction, which is not biocompatible and required several hours to complete.

We believe that our microfluidic workflow for microgel production shows great promise in microgel production for a number of reasons. First, the ruthenium cross-linking strategy is fast, efficient, and widely applicable to a range of proteins and could, for example, be adapted to create microgels from common protein biopolymers such as fibrin,^22^ gelatin,^25^ and silk.^32^ Second, although it is not the only available visible light cross-linker, it has key advantages over other strategies such as lithium phenyl-2,4,6-trimethylbenzoylphosphinate (LAP). In particular, it has a comparatively^13^ high absorptivity (ε ≈ 14 600 M^–1^ cm^–1^ at 450 nm), allowing lower reagent concentrations with less adverse effects on viability in cell culture applications.^13^ Finally, our approach could be adapted to proteins whose mechanics respond to chemical^33^ or mechanical stimuli.^21^

## Conclusions

We here developed a microfluidic workflow for producing microgels using visible light-driven photochemical cross-linking. The workflow consists of a flow-focusing droplet microfluidic device followed by a photochemical cross-linking area where the droplet cross-links into microgels under visible light. We manufactured spherical BSA droplets across the three different microfluidic devices employed. We observed that an increase in the Capillary number of the continuous phase *Ca*_c_ led to the formation of smaller droplets and a larger droplet generation rate. We also observed that the microgels retained their shape and size during photochemical cross-linking.

## Supporting Information Available

Viscosity curve of BSA solution with and without cross-linking agent (Figure S1); and elastic modulus of BSA hydrogels during gelation (Figure S2) (PDF)
