# Supplementary material for: A New Approach for On-Chip Production of Biological Microgels Using Photochemical Cross-Linking
Source: Anal Chem. 2024 Jun 11;96(25):10140–4. doi: 10.1021/acs.analchem.4c01574 (PMC11209654; doi:10.1021/acs.analchem.4c01574)
Supplement: Supplementary file 1 — ac4c01574_si_001.pdf [file ac4c01574_si_001.pdf]

Supporting Information
A New Approach for On-Chip Production of Biological Microgels using Photochemical Crosslinking

Francesco Del Giudice ,[†] Dan J. Curtis,[†] and Anders Aufderhorst-Roberts[*,‡]

†*Complex Fluids Research Group, Department of Chemical Engineering, School of Engineering and Applied Science, Faculty of Science and Engineering, Swansea University, Swansea, SA1 8EN, UK*

‡*Centre for Materials Physics, Department of Physics, Durham University, Durham, DH1 3LE, UK*

E-mail: a.aufderhorst-roberts@durham.ac.uk

Contents

Figure S1: BSA Viscosity curve with and without cross-linking agent S2

Figure S2: Elastic Modulus of BSA Hydrogels during Gelation S3

Figure S1: BSA Viscosity curve with and without cross-linking agent

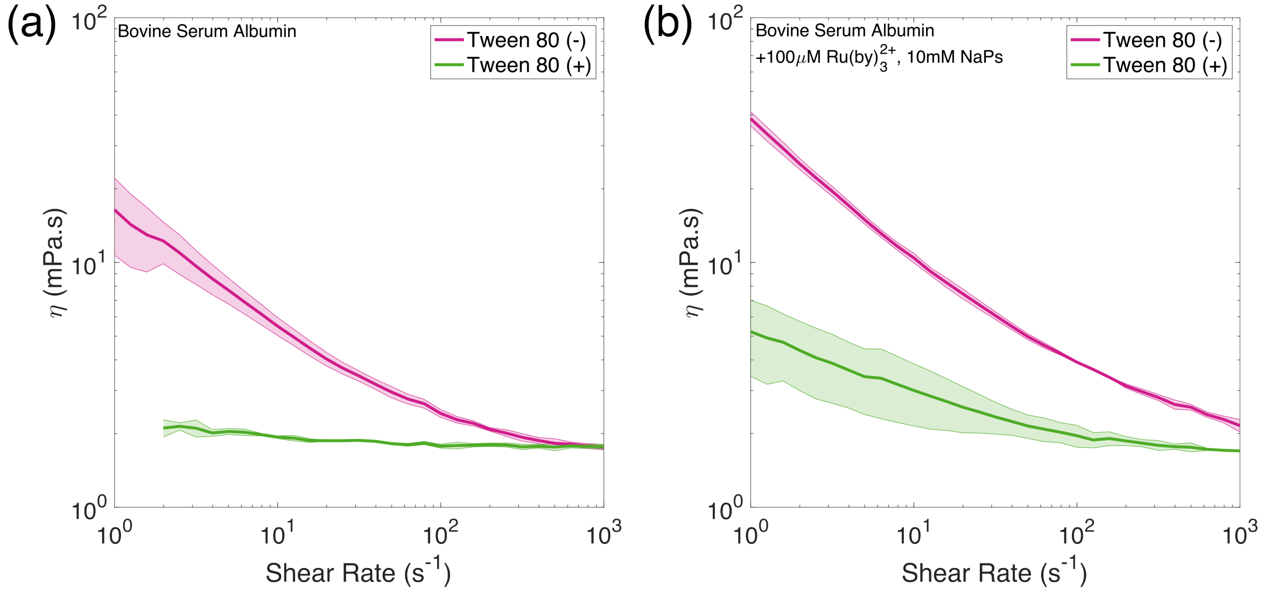

Fig. S1: Bulk steady shear viscosity of BSA solutions with respect to shear rate prepared at the concentrations used for microgel production in the presence (a) and absence (b) of crosslinking reagents. Although both samples show apparent shear thinning, the addition of 0.01% Tween-80 surfactant to either sample is shown to markedly decrease this apparent thinning behaviour, indicating that the apparent thinning arises from interfacial effects.

Figure S2: Elastic Modulus of BSA Hydrogels during Gelation

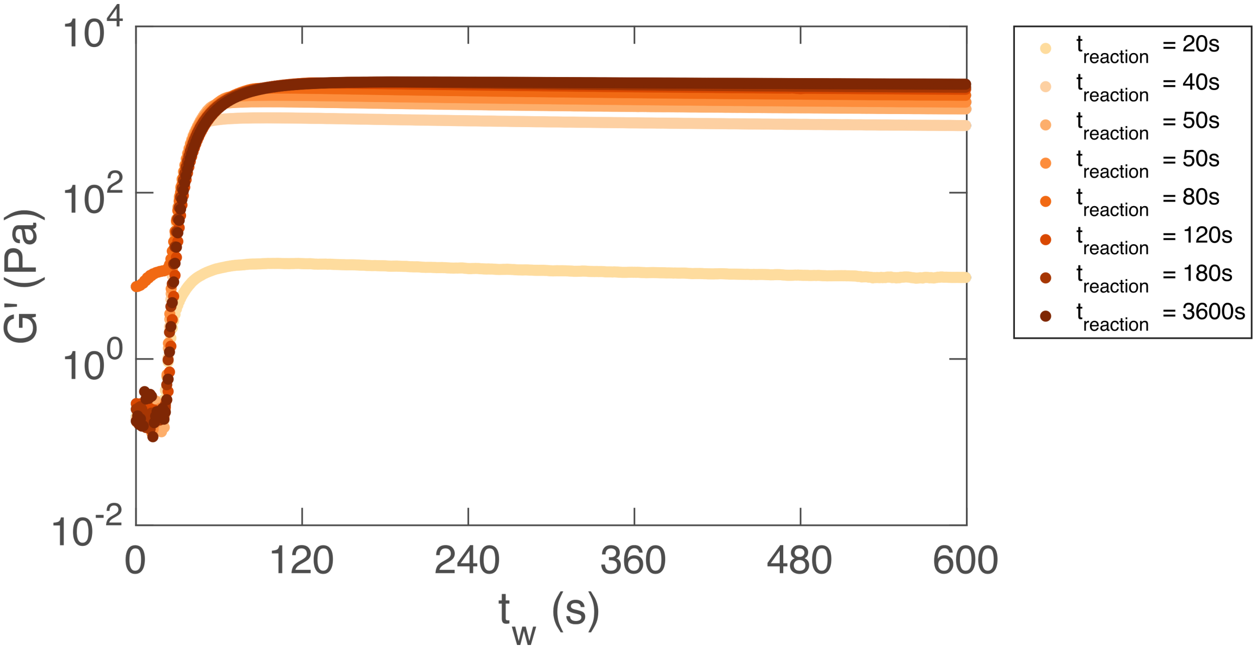

Fig. S2: Elastic modulus G' with respect to incubation time t_w of BSA following the initiation of photochemical crosslinking (a), for a range of reaction times $t_{reaction}$.
